# Dynamics in Transcriptomics: Advancements in RNA-seq Time Course and Downstream Analysis

**DOI:** 10.1016/j.csbj.2015.08.004

**Published:** 2015-08-24

**Authors:** Daniel Spies, Constance Ciaudo

**Affiliations:** a Swiss Federal Institute of Technology Zurich, Department of Biology, Institute of Molecular Health Sciences, Zurich, Otto-Stern Weg 7, 8093 Zurich, Switzerland; b Life Science Zurich Graduate School, Molecular Life Science Program, University of Zurich, Institute of Molecular Life Sciences, Winterthurerstrasse 190, 8057 Zurich, Switzerland

**Keywords:** RNA-seq, Time course analysis, Bioinformatics, Transcriptomics, Differential gene expression, Clustering

## Abstract

Analysis of gene expression has contributed to a plethora of biological and medical research studies. Microarrays have been intensively used for the profiling of gene expression during diverse developmental processes, treatments and diseases. New massively parallel sequencing methods, often named as RNA-sequencing (RNA-seq) are extensively improving our understanding of gene regulation and signaling networks. Computational methods developed originally for microarrays analysis can now be optimized and applied to genome-wide studies in order to have access to a better comprehension of the whole transcriptome. This review addresses current challenges on RNA-seq analysis and specifically focuses on new bioinformatics tools developed for time series experiments. Furthermore, possible improvements in analysis, data integration as well as future applications of differential expression analysis are discussed.

## 1 Introduction

Profiling of gene expression via high-throughput methods has been achieved for the first time in 1992 with the development of Differential Display protocols [1] followed in 1995 by the implementation of complementary DNA microarrays [2]. Subsequently, several other large scale techniques were developed like Serial Analysis of Gene Expression (SAGE) [3], Massive Parallel Signature Sequencing (MPSS) [4], Cap Analysis Gene Expression (CAGE) [5] and tiling arrays [6]. Finally, the breakthrough of RNA-seq [7] technology now offers scientist greater power, lower costs and new tools to better understand a wide spectrum of scientific and complex medical problems [8].

RNA-seq allows the assessment of the whole transcriptome (known and novel transcripts), including: allele specific expression, gene fusions, non coding transcripts such as long non coding RNAs (lncRNA), enhancer RNAs (eRNA) and the possibility to detect alternatively spliced variants (reviewed in [9,10]). Compared to microarrays approach, RNA-seq data is highly reproducible and allows the identification of alternative splice variants as well as novel transcripts [11]. Expression or tiling microarrays and capture arrays are still used intensively in biology and medicine for specialized tasks and diagnosis [12] due to the standardized protocols and gold standard bioinformatics analysis.

Several RNA-seq protocols for differential expression or detection of novel transcripts have been developed and can be classified into two main methods: enrichment of messenger RNA (mRNA) or depletion of ribosomal RNA (rRNA). For eukaryote genomes, the most common and so far standardized protocol is the selection of poly(A +) transcripts (mRNA) via oligo-dT beads enriching non rRNA fractions. The second category consists of the depletion of ribosomal RNA [13]. Several of these protocols, have been compared and reviewed in regards to different applications [14,15].

When studying dynamic biological processes [16] such as development or drug responses, datasets have to be captured continually in a Time Course (TC) experiment. Therefore, these data are sampled at several Time Points (TP) in order to recapitulate the whole regulatory network involved, identifying possible regulators and genes switches responsible e.g. for cyclic behavior or correct differentiation of cells. TC experiments can be classified into three groups [17]:

i) Single-time series investigating only one condition. Here, all time points are compared to the first one, which is considered as control. This approach requires fewer samples, but will not properly control for e.g. varying temperature in the incubator, as the control was not sampled over time.
ii) Multi-time series accessing several conditions simultaneously. The TC data sets are compared to a control TC. This approach allows to better control the experiment, due to the fact that controls are sampled over the time in parallel across the samples. Alternatively, the comparison can be performed directly between the different condition TCs. The drawback of this approach is higher costs, as more samples have to be sequenced and analyzed.
iii) Periodicity and cyclic TC consisting of single or multiple time series. A cyclic event of interest (e.g. cell cycle of proliferating cells) is investigated for reoccurring expression patterns and their differences between conditions. As at least two full cycles should be sampled for each condition, a large number of total samples are required to perform such experiments. Furthermore, differentiating between phases within the cyclic event might be challenging and may lead to “mixed datasets” due to non-uniform cell identities of mixed cell populations. Therefore, synchronization of cells prior the experiment is of importance to avoid “mixed datasets”.

As the complexity of the obtained data is increased by at least one dimension per TP of each sample, specific algorithms and methods are required to analyze TC experiments. Some have already been successfully implemented for microarray data. However, only few have been adapted for RNA-seq data (reviewed in [18]).

In the following sections of this review, we will discuss current challenges and available methods as well as promising improvements and extensions of RNA-seq Time Course experiments.

## 2 Methods

**Fig. 1:**
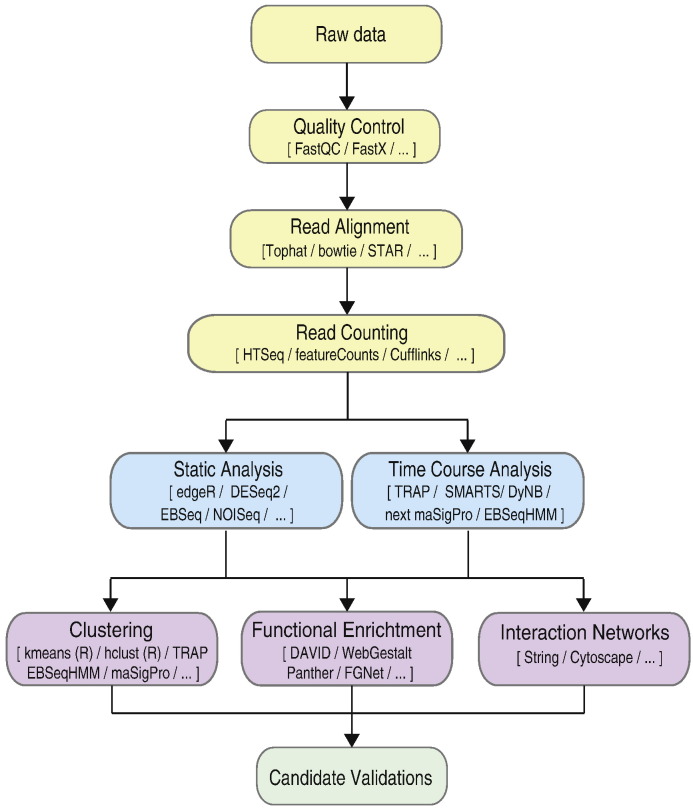
RNA-seq analysis workflow.

Time course experiments follow the same workflow as static RNA-seq experiments, starting with preprocessing and normalization of the data, followed by differential gene expression (DEG) and downstream analysis by clustering and network construction (Fig. 1).

In this review, we are only considering the analysis of RNA-seq TC data, therefore assuming that the data was already pre-processed (quality controlled, mapped and if necessary read count files created). We only consider whole population RNA-seq data, not including single cell RNA-seq approaches. For a complete overview and comparison of sequencing platforms as well as available tools for mapping reads the reader is referred to [19,20].

### 2.1 Biases/Challenges

#### 2.1.1 Experimental Design

Well known biases, such as GC content, gene/fragment length or batch effects [19] are currently assessed during the quality control step using QC tools like FastQC (available online under http://www.bioinformatics.babraham.ac.uk/projects/fastqc). Time course experiments introduce additional experimental and computational challenges that have to be addressed and will be further discussed.

As in other sequencing experiments, the experimental design is of utmost importance. Setting the sampling rate by defining the number of replicates per time point (TP) and the number of TP is still dictated by relatively high sequencing costs. In the case of microarray experiments, under-sampling has been shown to cause aggregation of effects due to insufficient temporal resolution [21]. Some tools are already available to facilitate sample size calculation for RNA-seq data [22,23]. These methods calculate a sample size of 20 to 79 or between 8 and 40 in order to detect differential expression (for the detection of a log fold change of 2 and power of 80%). However, such number of samples is for several experiments not feasible and most of these approaches do not consider multi-factor experiments. Recent estimations of power and sample size for RNA-seq have been performed on different datasets. This work revealed that 10 replicates on a 10,000$ budget restrain already yield maximum predictive power, a number of replicates that nevertheless could be still to high for static and especially time course experiments [24].

Moreover, choosing a feasible method to analyze data is depending on the experimental setup. This depends on whether it is a long or short time course (< 5 TP) experiment, or whether the time course was sampled uniformly and on how many replicates are needed for reliable and robust final statistics evaluation. Depending on the system investigated, it might also be necessary to synchronize the data in order to accomplish a uniform starting point to exclude phase (e.g. cell cycle, development, circadian rhythm) or patient specific (e.g. age or diseases) differences and therefore improve normalization and DEG analysis.

So far no gold standard method is established for RNA-seq data analysis, though for some specific applications guidelines have been recently published [25]. The sequencing depth is usually not posing a problem (unless when rare or novel transcripts have to be detected, which require a 100–200 × coverage for human or mouse genomes). A protocol of 100 bp paired end library preparation coupled with a minimum of three replicates should be established as minimum requirement for powerful statistics of DEG analysis [26]. When having to make a trade-off between sequencing depth and biological samples, Liu and colleagues showed that adding more replicates is increasing predictive power of detecting DEGs to a greater extend than sequencing depth [27].

The quality of the raw data is of importance for the subsequent bioinformatics analysis. Therefore, a good experimental design including a statistically relevant number of controls and replicates are essential for the quality control, mapping and normalization steps. Erroneous designs, including no replicates, will result in less powerful statistics, an increase of false positive candidates and will cause unnecessary and enormous costs in downstream analysis and validation experiments. Possible attempts to improve data quality are mentioned in the discussion of this review.

#### 2.1.2 Analysis

Several methods/tools have been developed for microarrays (e.g. lumi [28], affy [29]) or static RNA-seq (e.g. edgeR [30] or DESeq2 [31]) analysis. The most recent tools are able to solve the problems of differences in sequencing depth (library size), outliers and batch effects introduced by library preparation protocols, sequencing platform and technical variability between sequencing runs [32]. Even if some tools developed for static experiments can be used for TC data, one major issue is that they do not consider correlations of genes between previous and subsequently TP. Indeed, random patterns, overall time trends in expression or time shifts are therefore not taken into account for normalization, noise correction and differential expression steps. For example, a drug treatment could induce a slower metabolism of a cell population, resulting in a delay or change in the establishment of gene expression patterns. Such delay effects can be recognized only when using all TP data for analysis.

### 2.2 Differential Gene Expression Methods for Static RNA-seq Data Analysis

Most established methods for DEG analysis are parametric using count-based input and apply their own normalization approaches to raw data. The majority of parametric methods apply a negative binomial model to the read counts in order to account not only for the technical variance but also address the biological variance. Previously, Poisson distributions [11] were used to correct for the technical variance. The one-parameter distribution is not able to describe biological variance, which is higher than a calculated mean expression making the Poisson distribution unsuitable. Therefore a negative binomial distribution is used, adding a dispersion parameter to be more flexible accounting for biological variance and appropriately identifying DEGs [31,33,34].

Several non-parametric methods like NOISeq [35], or more recently NPEBseq [36] and LFCseq [37] offer an alternative way to normalize and model expression data, which are not fitting with negative binomial or Poisson distributions. Nevertheless, these methods are usually more computationally exhausting and need a higher number of replicates to perform equally well [26,38].

Major methods perform equally well in normalizing the data [39], but show significant differences in the number of DEGs identified, in accuracy and in power. In this review, we will not discuss each method in detail and we will not make a statement regarding which method to use. These methods were designed for a specific context and might be more appropriate for certain experiments. In conclusion, there is no overall best method for all types of analysis. However, we would like to emphasize the importance of considering the following aspects when choosing a method for analyzing the data to meet the experimental design:

- How many replicates are needed for this method?
- Is a simple two-way comparison sufficient or is a more complex multi-factor model needed for DEG analysis?
- Is it desirable to detect differentially expressed RNA isoforms as well?

### 2.3 Differential Gene Expression Methods for TC RNA-seq Data Analysis

Time Series experiments have been extensively conducted in the past using microarrays, providing algorithms such as spline fitting [40,41], Bayes statistics [42,43] or Gaussian processes [44,45] to account for temporal aspects of DEG. Moreover, algorithms detecting periodic patterns have been also developed (e.g. Lomb–Scargle periodograms [46]). Most of them have been implemented into pipelines such as STEM [46], maSigPro [47], BETR [48], TIALA [49] and platforms for researchers like PESTS [50].

To date, there are only five tools available to implement RNA-seq TC data for DEG analysis that we would like to describe in more detail (Table 1. Of Note, more detailed explanations of standard statistic models and tests can be found in text books [51,52] and detailed information about new approaches are described in the corresponding literature cited).

Next maSigPro [53] is an updated version of maSigPro, an R package on Bioconductor (http://www.bioconductor.org) initially developed for microarray TC experiments. The updated version allows the analysis of RNA-seq TC data as well. It uses generalized linear models instead of a linear model in order to allow the modeling of count data. This is achieved by fitting to a negative binomial distribution followed by a polynomial regression. In order to be detected as DEG, the difference of Log Likelihood Ratio of the hypotheses has to be greater than a user defined significance threshold. This ensures a best-fit model for each gene by only keeping significant coefficients. Though, Next maSigPro does not offer built-in normalization methods, the package is equipped with functions for clustering and visualization of processed data.

In a comparison with edgeR package, Next maSigPro can control better the False Discovery Rate (FDR). Candidates identified by both approaches or solely by Next maSigPro have highly significant and well-fitted models, while the majority of the candidates selected only by edgeR do not pass the second significance threshold step. The small number of DEG not pre-selected by Next maSigPro has a high variance as well as a little fold change. One first drawback of the pipeline is that the threshold for DEG detection is not set automatically according to the data but it is a user defined threshold, making it more challenging to indirectly determining a FDR. Furthermore, the user has to define the number of clusters, whereas it would be better if the number of clusters would be determined based on the actual data. Finally, replicates are not merged with error bars in the output graph but data points are plotted one after each other.

DyNB [54] uses negative binomial likelihood distribution to model count data taking a temporal correlation of genes into account. It is also correcting for time shifts between replicates and time-series by Gaussian processes introducing time-scaling factors. Normalization is performed by variance estimation and rescaling of counts similar to DESeq [55], but on the previously calculated Gaussian process function rather then directly on the samples. In the next step DyNB uses a Markov-Chain-Monte-Carlo (MCMC) sampling algorithm for marginal likelihoods that enables the DEG analysis. A comparison of the DyNB and DESeq candidates showed that the DyNB outperforms DESeq for the detection of weakly expressed or high noise level genes as well as genes affected by variable differentiation efficiency. A drawback is the implementation in MATLAB® (The MathWorks Inc.), thereby making it less accessible to a broad range of users. Additional drawbacks are: long running times due to MCMC sampling; genes not expressed in one condition are removed; the test output is a Bayes factor calculated by the ratios of hypothesis probabilities, which is less intuitive than the more common p-value. Finally, according to Jeffreys et al. [56], a Bayes Factor value higher than 10 is referring to a strong evidence of differential expression, though this threshold might not hold true for all types of datasets and users will have to adapt filtering to identify their candidates of interest.

TRAP's [57] is a method that aims to identify and analyze differentially activated biological pathways. In a first step, reads are mapped to a reference genome by the Tophat [58] software and further processed to estimate the expression by Cufflink [59]. In the second step, the DEG analysis is performed by the Cuffdiff software [60], generating a FPKM (“reads per kilobase of transcript per million reads mapped”) output file for each sample. The novelty is the downstream analysis, by directing DEG candidates from the Tophat/Cufflinks/Cuffdiff pipeline into a KEGG analysis [61,62]. This approach offers three options: One Time Point pathway analysis, Time Series pathway analysis or Time Series clustering. The one time point analysis identifies significant pathways for each time point separately, whereas the Time Series pathway analysis takes all TP into account. For pathway analysis two methods are performed and their p-values combined: Over-representation Analysis (ORA) using the Gene Ontology (GO) [63] database and a Signaling Pathway Impact Analysis (SPIA) [63]. Briefly, ORA identifies significant pathways by hyper-geometric tests that compares the ratios of DEGs to the complete number of genes on a total and pathway level. SPIA takes the effect of other genes in a pathway into account. This is achieved by calculating a perturbation factor of fold change of upstream genes divided by the fold change of downstream genes. Additionally, it introduces a time-lag factor for Time Series analysis.

For Time Series Clustering, each gene is assigned to a label at each time point, depending on whether the log-fold change of FPKM is either positively/negatively above a threshold or otherwise categorized as constant. Clusters are generated by grouping genes with the same label and further analyzed by ORA using ratios of pathway genes to total genes and all genes in the cluster. Users can directly start the downstream analysis by providing Cufflink/Cuffdiff data avoiding the time demanding preprocessing steps. The main pipeline is performing a pairwise comparison of TPs. Of notes, it is not making use of the time series parameter of Cuffdiff, but only takes the temporal character in later analysis into account. For the analysis itself, a possible complication is the conversion of gene name Identifiers to match the ones used in the pathway files. Moreover only the first of possible several gene name identifiers for a given pathway is used to find matches among candidates. In our opinion, the major drawback of the pipeline, similar to DyNB, is that the genes that are not expressed in one condition are excluded from further analysis. This is due to an infinite log fold change ratio caused by non-expressed genes, which are assigned zero as expression level.

SMARTS [64] is designed to create dynamic regulatory networks based on time series data from multiple samples by iteratively creating models extending the DREM method [65]. First, samples are synchronized to a common biological time scale by pairwise alignment followed by sampling of points. This allows a continuous representation, correction of alignment parameters and a computation of an error metric in order to create a weighted alignment. A second alignment error is calculated between samples to create a matrix for an initial clustering by spectral clustering or affinity propagation for cases with two or more clusters, respectively. Clustering is calculated on the basis of all genes and contains noise. SMARTS takes advantage of the fact that a certain condition is only affecting a small number of genes that are regulated by an even smaller number of transcription factors (TFs) and up-stream pathways. This in turn, reduces the dimensionality of the data. The clustering is the basis for a first regulatory model that is iteratively adapted to create a final clustering of groups that are co-expressed and regulated throughout the time-series. To iteratively improve the regulatory models, static protein–DNA interaction data, such as DNA-binding motifs or ChIP-seq data, is used to define the path of each gene by modeling the transition between time points applying an Input–Output Hidden Markov Model framework. The regulatory model converges into a final clustering that identifies split time points where a subset of genes that have previously been co-expressed diverge into another path. The resulting graph offers a view of gene sets and their path throughout the timeline illustrating the differences in TF at splits that are most likely responsible for the differences in expression and regulation of subsequent time points. In our opinion, the only drawback of this tool is the requirement of prior knowledge of TF binding to genes of interest used as input to the pipeline.

EBSeq-HMM [66] is an extension of the EBSeq package [67] accounting for ordered data (e.g. such as time, space, gradients) by applying an auto-regressive Hidden Markov Model (HMM). EBSeq-HMM identifies dynamic processes (genes that are neither unchanged nor sporadically expressed) and classifies genes according to their state (up/down/unchanged) into expression paths taking dependencies to prior time points into account. The analysis is based on two steps: first, the conditional distribution of data at each time point followed by the transition of states over time. Parameter estimation for the conditional distribution is performed using a beta-negative-binomial model. Second, an additional implementation to correct for the uncertainty of read counts of genes with several isoforms is offered. Subsequently, a state for each gene at each time point is determined applying a Markov-switching auto-regressive model to account for the dependencies of expression and state of the previous state. Finally, all the states of a gene are combined and classified into an expression path.

The developers also tested EBSeq-HMM together with existing static methods and Next maSigPro on simulated and case study data. On the simulated data EBSeq-HMM performed with greater power and F1 scores (a score to access a test's accuracy) but had a higher false discover rate (FDR) of 4.5% in comparison to a maximum of 0.5% compared to the other methods. On clinical data, EBSeq-HMM had a 90% overlap of identified genes with other methods and outperformed these on genes with subtle and consistent changes over time. However, the authors did not make any statement about the genes, which EBSeqHMM was not able to identify. When using EBSeqHMM, the user has to keep in mind that its purpose is to identify dynamic genes; in theory it also identifies constant genes and clusters them accordingly. Practically, in order to be constant, the previous and following TP have to have the exact same mean expression value, resulting that most genes will be classified as up or down regulated at affected TPs and hiding possible non DEG time intervals of genes.

### 2.4 Downstream Analysis

DEG analysis may result in hundreds of putative candidates, if not more, a number that cannot be experimentally validated. Therefore, scientists tried to reduce the number of candidates by searching for expression patterns and shared pathways to narrow down essential candidates. This field has been extensively researched and improved over the last two decades offering a great abundance of tools, leading to new scientific questions and simplifying their validation.

#### 2.4.1 Clustering Methods

The purpose of clustering is to statistically group samples according to a certain treat, e.g. for gene expression, to reduce complexity and dimensionality of the data, predict function or identify shared regulatory mechanisms. Depending on the data structure a fitting clustering method has to be used to account for the specific data (reviewed in [68]). Considerations should include:

- Was the data transformed or does it consist of read counts?
- How is it distributed?
- Is the data originating from static, short or long TC experiments?

A plethora of clustering methods have been published, many of them available as R packages on the CRAN Task View page (http://cran.r-project.org/web/views/Cluster.html), the Bioconductor website (http://www.bioconductor.org) or in other scripting/programming languages made available on the publishers' web sites. However, we cannot discuss the whole spectrum of these methods. Therefore, we would like to point out certain methods which are specific for TC experiments employed for microarray [69–71] and RNA-seq data [72,73] and refer to the afore mentioned reviews for the selection of a fitting method.

#### 2.4.2 Functional Enrichment Analysis and Network Construction

To gain new insights into complex data, one of the most common methods used is functional enrichment analysis (FEA). FEA identifies candidates sharing biological function or pathway by statistical over-representation using annotated databases such as Gene Ontology [63] or KEGG [61,62] and can easily be performed using available free web interfaces or R packages such as DAVID [74], WebGestalt [75], PANTHER [76] or FGNet [77], Finally, several commercial software also exist, such as Ingenuity [78] or Metacore [79]. Other options are the investigation of direct and indirect protein–protein interactions via the STRING database [80] or via Cytoscape applications [81]. Detailed descriptions, comparison and overview of FEA tools can be found in recently published reviews [82–84].

### 2.5 Discussion

In the last few years, many algorithms were developed to increase the quality and methodology of existing approaches. A usual procedure is to extend, adapt or update an existing established method. For example, edgeR was updated by multifactor experiments [85] and observation weights factor [34] to more robustly account for outliers. Combining existing methods and new strategies could offer a great improvement in quality of analysis, in static as well as in TC experiments.

Here, we present novel advancements in the field that might offer improvements to existing methods and pipelines. Major issues at the level of mapping and the quantification of reads are: ambiguous (overlapping genes), multi-alignment (repeats) and exon-junction reads, which are usually discarded at the counting step. Recent approaches such as GIIRA [86], ORMAN [87] and Rcounts [88] account for multi-mapping reads by introducing a maximum-flow optimization, minimum-weighted set cover problem of partial transcripts and weighting alignment scores, respectively. These recent improvements allow a better quantification of genes and isoforms, as well as the investigation of repeat elements, which was up do date not very feasible. On the isoform level, WemIQ [89] applies a weighted-log-likelihood expectation maximization for each gene region separately to improve quantification of isoforms and gene expression.

Samples that differ highly in read counts (extreme high counts) create a bias at the normalization step due to the adjustment to a common scale that is calculated over all samples. This problem is addressed by the RAIDA algorithm [90], which accounts for differences in abundance levels rather than modifying the read counts for normalization. Further studies of the SEQC/MAQC—III Consortium elucidated the negative influence of lowly expressed genes on the DEG detection [19,91,92]. Therefore, filtering out genes with low expression might offer another possibility to increase predictive power.

Another problematic aspect in analysis arises when working with small sample size (less than 4 replicates per TP). In such cases, for RNA-seq experiments, the calculation of the dispersion factor of negative binomial methods is less accurate. Therefore, a new shrinkage estimation [93] has been introduced in order to analyze data with few replicates (4 or less), which was incorporated into a new tool sSeq [33]. Moreover, resampling of at least three biological replicates per time point was shown to improve the identification of oscillating genes without increasing false positives rates [94]. Recently, a new adapted exact test has been developed to increase power in order to detect DEGs for experimental designs containing only two replicates. This R package is also able to identify differentially expressed genes that are not abundant [95].

As there is no best fitting method for DEG analysis so far, we recommend using several tools and compare and combine the results in order to obtain confident candidates. To increase precision, sensitivity and reduce the detection of false positives candidates, a combination of statistical tests should be applied. The PANDORA algorithm [96] combines p-values, using one of six possible methods, which have been weighted based on the performance of each statistical test. On the other hand, multiple testing and combination of results involve an increase in time and resources needed to run the analysis, which might outweigh the gain in the power of the statistics. In the beginning of multi-Omics analysis, RNA-seq data was used to improve results of other approaches when the initial method reached it limits. With further advancement and availability of technologies, scientists started to combine several Omics data to ask new scientific questions and to add additional layers of information to their data. Further, a great increase and expansion of databases such as ENCODE [97], Cancer Genome Atlas (http://cancergenome.nih.gov), GEO [98], KEGG [61,62] and analysis platforms have also facilitated the access to multi-Omics analysis. Nevertheless, the integration of several Omics datasets still harbors several challenges such as quality assurance, data/dimension reduction and clustering/classification of combined data sets [99], which have to be properly addressed and taken into account when designing experiments and performing analysis. In the following paragraph we would like to highlight methods that combine static or TC RNA-seq experiments with other Omics data. These tools can be categorized on whether they are multi-staged or meta-dimensional approaches, performing different Omics analysis sequentially or combining several data types into a single analysis [99,100].

In the past decade, great efforts were undertaken to develop and improve tools combining microarrays and ChIP-seq data (e.g: ChIP Array [101], EMBER [102] for static experiments, and for TC experiments [103,104]). Up to date, there are several multi-stage tools available to analyze RNA-seq and ChIP-seq, e.g. INsPeCT [105] and metaseq [106], but only few integrated meta-dimensional approaches e.g. Beta [107], CMGRN [108] and Ismara [109]. Nevertheless, none of the mentioned methods offer specific TC algorithms for analysis, and most tools either aim to identify targets of transcription factors (TFs) and create Gene Regulatory Networks (GRN), whereas others use methylation or histone modification data to predict regulatory functions [110].

Different approaches and tools for the integration of other Omics data have been extensively reviewed for proteomics [111], metabolomics [112] and phenotypic data [113]. Indeed, re-analyzing externally obtained data using the same pipelines used for in-house produced data sets is the best approach in order to guarantee comparable results.

In general, more powerful algorithms, which so far have not been implemented due to technical infeasibilities, become more and more available. Nevertheless, the optimization through parallelization and cloud computing is a major goal for the development of such new tools. As the amount of data produced in each experiment is massively increasing, improved pipelines and algorithms are in demand in order to supply the users with a good trade-off between accuracy and resources needed for their analysis.

## 3 Conclusion and Perspectives

Recently, two approaches emerged, namely co-expression analysis and single cell RNA-seq, that are very promising to improve DEG analysis and offer new application fields such as the study of subpopulations.

The assumption behind co-expression analysis is that genes in the same pathway very likely share regulatory mechanisms and therefore should have the similar expression patterns. This allows the identification of biological entities that are involved in the same biological processes and has already successfully been applied to microarray data [114]. Moreover, microarray co-expression data has been also integrated with other data types such as microRNA [115] or phenotypic [116] data and been used for differential co-expression to identify biomarkers [117]. It has further been shown that co-expression analysis is able to improve sensitivity of RNA-seq DEG analysis [118] and more recently to outperform existing clustering approaches [119]. Similarities and differences of co-expression networks in microarrays and RNA-seq as well as factors driving variance at each stage of co-expression analysis have already been investigated [120]. However, no gold standard for RNA-seq co-expression analysis has been established.

Single-cell RNA-seq, in contrast to population sequencing, enables to access the heterogeneity of gene expression in cells which otherwise is averaged out or even lost for small subpopulations of cells in bulk experiments. This heterogeneity in expression arises due to differences in kinetics of response to a certain condition, treatment or cell fate decisions of each cell. Single-cell RNA-seq allows studying the subpopulation of interest and investigating mechanisms explaining differences between subpopulations, which might offer advances in drug development, personalized medicine or the creation of differentiation networks. Improvement in protocols and sequencing lead to new methods at a rapid rate: STRT [121], CEL-Seq [122], Smart-seq [123], Quartz-seq [124] and microfluidic platforms [125], enabling scientists to ask new questions. Nevertheless, protocols and methods for single-cell sequencing are not yet completely optimized and still harbor uncertainties such as noise, sequencing and normalization biases as well as proper tools for analysis. There is great effort to address these problems. It has been recently reported that explicit calculation of gene expression levels using External RNA Controls Consortium spike in controls [126,127] improved normalization and noise reduction [128]. Finally, up to date the lack of validated genome-wide data slows down the development of new algorithms and models can only approximate the real extent of regulation or networks [129]. There are tools to simulate expression data incorporating noise, such as SimSeq [130], but still this noise estimation does not completely capture a biological situation and again is just an estimation of the whole picture. As more and more genome-wide experiments are conducted, networks created and candidates validated, the data of several sources could be compiled into a database offering frameworks for model validation.

To conclude, in the last decades a plethora of new models, system and networks were created, with the caveat of over-generalization of results in order to fit hypotheses and models. By combining high-throughput data, scientists are now able to correct for this over-generalization by filling gaps with complementary data, allowing fine-tuning and dissection of existing models and networks as well as the upcoming of new intuitive, integrative and explorative tools. Further, the integration of several kinds of Omics data remains the biggest challenge [131] as we have to understand the limitations of each technique before conducting a joint analysis [111] and to develop several tools according to the specific data types and underlying genomic models for powerful integrative analysis [99].

## Figures and Tables

**Table 1:** Properties of available time course analysis tools: ^a^ negative binomial model, ^b^ polynomial regression, ^c^ log likelihood ratio, ^d^ gaussian process, ^e^ marginal likelihood, ^f^ Markov Chain Monte Carlo, ^g^ over representation analysis, ^h^ pathway topology based analysis, ^i^ log fold change, ^j^ input output Hidden Markov Model, ^k^ randomization test, ^l^ auto regressive Hidden Markov model, ^m^ empirical Bayesian method. If a tool has several normalization methods, the standard method is underlined.

Method	Normalization method	Model	DEG test	FDR corr. p-values	Multi-factor experiment	Uneven TP allowed	Isoform detection	Clustering	Random pattern detection	Delay detection	Ref
Next maSigPro	—	NB^a^ + PR^b^	LLR^c^	Yes	Yes	No	No	Yes	No	No	[53]
DyNB	Variance estimation + scaling factors on GP	NB + GP^d^	ML^e^ by MCMC^f^	Yes	Yes	Yes	No	No	–	Yes	[54]
TRAP	FPKM/poisson quartile/geometric	ORA^g^ + PT^h^	LFC^i^	Yes	No	No	Yes	Yes	No	No	[57]
SMARTS	Pairwise weighted alignment	GP + IOHMM^j^	LLR + RT^k^	No	Yes	Yes	No	Yes	No	Yes	[64]
EBSeq-HMM	Median/quantile	beta NB + AR-HMM^l^	EB^m^	Yes	Yes	Yes	Yes	Yes	Yes	Yes	[66]
